# Acute Isolated Right Ventricular Infarction

**DOI:** 10.1016/j.jaccas.2023.102176

**Published:** 2023-12-20

**Authors:** Karim D. Mahmoud, Wijnand K. Den Dekker

**Affiliations:** Department of Cardiology, Thorax Center, Cardiovascular Institute, Erasmus Medical Center, Rotterdam, the Netherlands

**Keywords:** acute coronary syndrome, coronary angiography, electrocardiogram, myocardial infarction, myocardial revascularization, percutaneous coronary intervention

## Abstract

The electrocardiogram is universally used to diagnose ST-segment elevation myocardial infarction and serves as guidance for the interventional cardiologist to identify the acute thrombotic lesion. However, this case illustrates that the electrocardiogram can also be deceiving.

## Graphical abstract

**Figure:**
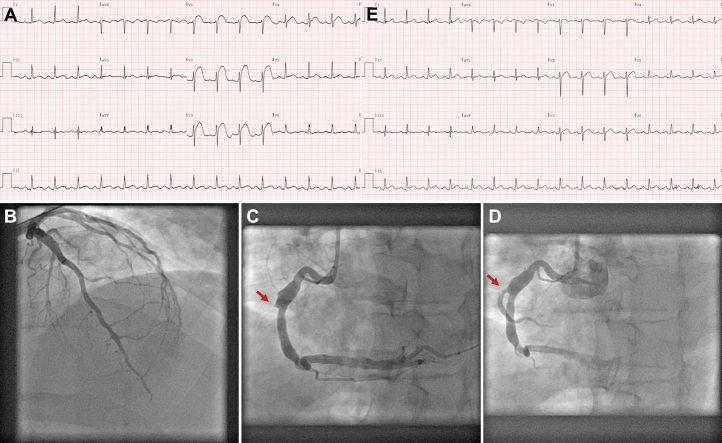

A 54-year-old man with a medical history of hypertension and intracranial bleeding contacted emergency medical services because of sudden-onset severe chest pain. The electrocardiogram showed ST-segment elevation in leads V_1_-V_4_ (Figure 1A). He had an episode of ventricular fibrillation requiring defibrillation during transportation. Upon admission, his blood pressure was 105/77 mm Hg, and his heart rate was 91 beats/min. Urgent coronary angiography surprisingly showed only wall irregularities in the left anterior descending artery (Figure 1B). Careful examination of the dominant ectatic right coronary artery revealed an ostial occlusion of a right ventricular (RV) branch. Intravascular ultrasound confirmed that it was an acute thrombotic lesion, which we successfully treated with a 3.0 × 15 mm stent (Figures 1C and 1D, Video 1). Residual thrombus at the ostium of the RV branch was noted on the final angiogram (Figure 1D, Video 1), which was treated with unfractionated heparin during 24 hours with a target activated partial thromboplastin time of 60 to 80 seconds. Glycoprotein IIb/IIIa inhibitors were deemed to be contraindicated in this patient with prior intracranial bleeding. The patient became asymptomatic, and the electrocardiogram normalized directly after the procedure (Figure 1E). Peak troponin was 684 ng/L (reference <14 ng/L), and peak creatinine kinase-MB isoform was 23.3 μg/L (reference <7.6 μg/L).

**Figure 1:**
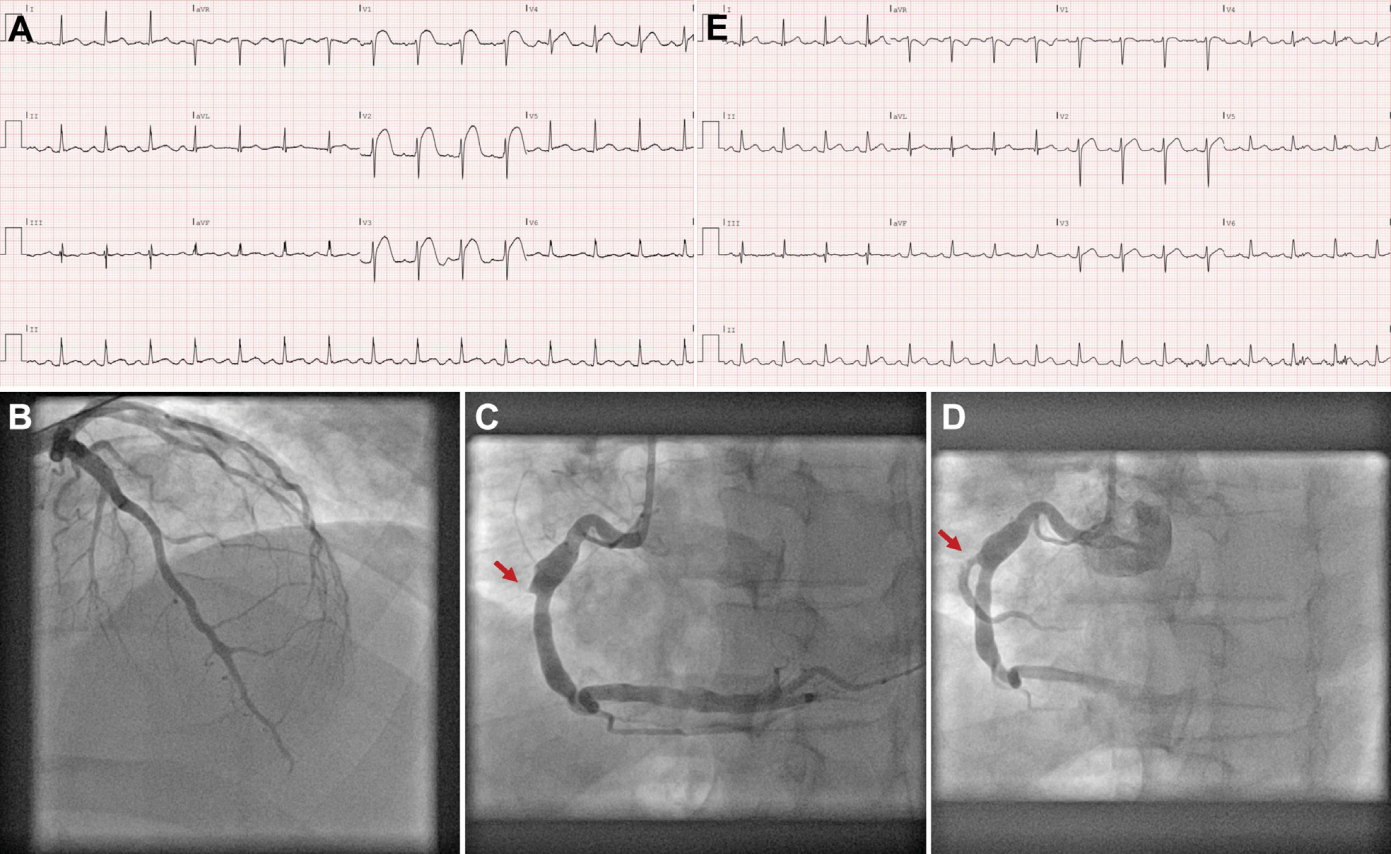
Acute Isolated Right Ventricular Infarction (A) Admission electrocardiogram showing ST-segment elevation in leads V_1_-V_4_. (B) Coronary angiogram of the left anterior descending artery showing wall irregularities only. (C) Initial coronary angiogram of the right coronary artery. The location of the occluded right ventricular branch is highlighted (red arrow). (D) Final coronary angiogram of the right coronary artery. The right ventricular branch is reopened with some residual thrombus at the ostium (red arrow). (E) Postprocedural electrocardiogram showing resolution of the ST-segment elevation.

Acute isolated RV infarction, especially due to occlusion of a RV branch in a dominant right coronary artery, is a rare condition that presents with ST-segment elevation in the anterior leads.^1^ The ST-segment elevation is usually the most pronounced in leads V_1_-V_2_, as these leads directly face the RV wall. ST-segment elevation can also be appreciated in the right-sided precordial leads, especially V_4_R. Acute isolated RV infarction is easily misdiagnosed, as ST-segment elevation in V_1_-V_2_ typically indicates involvement of the left anterior descending artery, and the angiographic image of an occluded RV branch can be very subtle. The patient was event free and asymptomatic at the 6-month follow-up.

## Funding Support and Author Disclosures

The authors have reported that they have no relationships relevant to the contents of this paper to disclose.

## Appendix

Video 1 Coronary Angiograms Coronary angiograms of the right coronary artery showing the occluded right ventricular branch on the initial angiogram. The second angiogram shows the final result. The right ventricular branch is patent again after stenting; some residual thrombus can be appreciated at the ostium of the branch.
